# Complete Genome Sequence of *Dolosigranulum pigrum* from a Patient with Interstitial Lung Disease Using Single-Molecule Real-Time Sequencing Technology

**DOI:** 10.1128/genomeA.00317-17

**Published:** 2017-05-04

**Authors:** Rituparna Mukhopadhyay, Joselita Joaquin, Robin Hogue, Susan Fitzgerald, Guillaume Jospin, Kristin Mars, Jonathan A. Eisen, Vishnu Chaturvedi

**Affiliations:** a Microbial Diseases Laboratory, California Department of Public Health, Richmond, California, USA; b Kaiser Permanente, Santa Rosa Medical Center, Santa Rosa, California, USA; c University of California Davis Genome Center, Davis, California, USA; d Pacific Biosciences, Menlo Park, California, USA

## Abstract

The whole genome sequence of *Dolosigranulum pigrum* isolated from the blood of a patient with interstitial lung disease was sequenced with the Pacific Biosciences RS II platform. The genome size is 2.1 Mb with 2,127 annotated coding sequences; it contained two clustered regularly interspaced short palindromic repeats (CRISPR)/CRISPR-associated proteins (Cas) systems.

## GENOME ANNOUNCEMENT

*Dolosigranulum pigrum* is a Gram-positive, catalase-negative, bacterium distinguished as a new genus among *Gemella*-like organisms from human infections (1). It is facultative, anaerobic, and commensal of the oral cavity implicated in several disease conditions rarely. We performed whole genome sequencing (WGS) of *D. pigrum* due to the lack of high-quality genomes in the NCBI database.

A Gram-positive coccus was isolated from the blood of an 82-year-old male. The patient had chronic hypoxemic respiratory failure due to interstitial lung disease, and had been hospitalized for pneumonia when the blood cultures that grew this organism were observed on blood-chocolate agar. The organism was identified as *D. pigrum* based upon conventional biochemical tests and 100% 16S rRNA gene sequence similarity to a *D. pigrum* isolate deposited in the NCBI GenBank database.

DNA from the bacterial culture was extracted using the Promega Genomic DNA Wizard kit. WGS was performed using Pacific Biosciences RS II Single-Molecule Real Time (SMRT) sequencing technology. A 10 kb library was prepared according to the manufacturer’s protocol using AMPure PB Beads (Pacific Biosciences, Menlo Park, CA). A total of 5 µg of genomic DNA was sheared using g-Tubes from Covaris (Woburn, MA). The DNA was concentrated, end-repaired, and ligated to hairpin adapters from PacBio. The loading concentrations for the libraries were 0.0075 nM and 0.0125 nM. The template was loaded into SMRT cell v3 using a Mag Bead kit. Sequencing was performed using two SMRT cells and a 360-min movie was acquired from each SMRT cell.

The sequence data were assembled by using the Hierarchical Genome Assembly Process 3.0 (HGAP 3.0) in SMRT Portal v2.3.0. After quality-filtering and trimming, 38,985 raw subreads were generated of an average length 11,676 bp totaling 455,194,279 bp. The genome was assembled into three contigs of total length 2,121,027 bp. The maximum contig length was 2,097,175 bp with an average coverage of 167.3×. The G+C content of the genome is 39.6%. BLASTn of the 16S rRNA gene sequence from the assembly against the NCBI reference RNA sequences (refseq_rna) database showed 99% similarity to *D. pigrum* strain R91/1468. The neighbor-joining phylogenetic tree analysis of 16S sequence showed that *D. Pigrum* isolate forms robust cluster with *D. pigrum* strain R91/1468 (NR_026098.1) and *D. pigrum* strain NCFB 2975 (X70907) (https://figshare.com/s/84e63b44517860fadd1d). The PHAST server predicted three prophage regions with sizes of 16.8 kb, 34.1 kb, and 36.6 kb (2).

Gene predictions and annotations were performed with the Rapid Annotation using Subsystem Technology (RAST) (3–5) database and Prokka version 1.1 (6). The annotation for the *D. pigrum* genome using RAST showed 300 subsystems, 2,127 coding sequences, and 74 RNA genes (https://figshare.com/s/84e63b44517860fadd1d). There were 2,236 genes, 17 rRNAs, 58 tRNAs, 1 tmRNA, and 2 repeat regions annotated by Prokka. Notably, the genome of *D. pigrum* contains Clustered Regularly Interspaced Short Palindromic Repeat (CRISPR) loci and Cas proteins (cas9, cas1, and cas2). The Cas proteins are RNA-guided DNA endonucleases associated with the CRISPR and constitute bacterial adaptive immune defense against viruses (7).

### Accession number(s).

This whole-genome shotgun project has been deposited at DDBJ/ENA/GenBank under the accession no. MUYF00000000. The version described in this paper is version MUYF01000000.
